# Risk of tumor lysis syndrome in patients with acute myeloid leukemia treated with venetoclax-containing regimens without dose ramp-up

**DOI:** 10.1007/s00277-020-04181-5

**Published:** 2020-07-23

**Authors:** Rabia Shahswar, Gernot Beutel, Razif Gabdoulline, Christian Koenecke, Dominik Markel, Matthias Eder, Michael Stadler, Gudrun Gohring, Brigitte Schlegelberger, Arne Trummer, Juergen Krauter, Felicitas Thol, Michael Heuser

**Affiliations:** 1 Department of Hematology, Hemostasis, Oncology, and Stem Cell Transplantation, Hannover Medical School, Carl-Neuberg Straße 1, 30625 Hannover, Germany; 2 Institute of Human Genetics, Hannover Medical School, Hannover, Germany; 3 Department of Internal Medicine, Municipal Hospital Braunschweig, Braunschweig, Germany

To the Editor,

The orally available BH3 mimetic venetoclax promotes apoptosis through selective inhibition of pro-survival protein B cell lymphoma 2 (BCL2) [1] and demonstrated single-agent activity and a tolerable safety profile in patients with relapsed or refractory acute myeloid leukemia (AML) [2]. Subsequent studies reported promising response rates of venetoclax combined with both low-dose cytarabine (LDAC) and hypomethylating agents (HMA) in untreated primarily elderly AML patients leading to its approval for newly diagnosed AML patients who are 75 years or older or who have comorbidities that preclude the use of intensive induction chemotherapy [3, 4]. Outside the US venetoclax is used off-label in relapsed/refractory patients with variable response rates [5]. In chronic lymphocytic leukemia (CLL), patients receive venetoclax in a weekly ramp-up dosage to mitigate the risk of tumor lysis syndrome (TLS) [6]. Despite the prophylactic use of uricostatic agents, TLS is of potential concern in venetoclax-treated AML patients, and only limited data on its clinical relevance in relapsed/refractory patients are available.

We report the incidence and outcome of tumor lysis syndrome in AML patients treated with full-dose venetoclax without dose ramp-up in combination with non-intensive chemotherapy for relapsed or refractory AML (*n* = 40) or newly diagnosed AML (*n* = 2) based on data reported to our venetoclax registry. Patients aged 18 years or older with acute myeloid leukemia as defined by the World Health Organization criteria [7], who had been treated with HMA or low-dose cytarabine combined with venetoclax and were reported to the venetoclax registry (venreg.org), were included in the analysis. Venetoclax was administered at a dose of 100 mg once daily perorally (days 1–14, in four patients days 1–28) due to mandatory co-medication with a CYP3A4-inhibitor for fungal prophylaxis. TLS was evaluated according to the Cairo-Bishop definition [8] and divided into laboratory TLS (LTLS) or clinical TLS (CTLS) (Supplementary Tables S1A and S1B). Biochemical values included potassium (hyperkalemia defined as potassium ≥ 6.0 mmol/L or ≥ 25% increase from baseline), calcium (hypocalcemia defined as calcium ≤ 1.75 mmol/L or ≥ 25% decrease from baseline), phosphate (hyperphosphatemia defined as phosphate ≥ 1.45 mmol/L or ≥ 25% increase from baseline), uric acid (hyperuricemia defined as uric acid ≥ 476 μmol/L or ≥ 25% increase from baseline), and creatinine (elevated creatinine levels defined as creatinine ≥ 1.5 times greater than the institutional upper limit of normal (ULN)). Clinical manifestations included seizure and cardiac arrhythmia/sudden death. All patients had given written informed consent to the off-label use of venetoclax, genetic analysis, and the use of clinical data according to the Declaration of Helsinki and institutional guidelines. The registry was approved by the local Ethics Review Committee (ethical vote No.7972_BO_K_2018) and is registered at https://www.clinicaltrials.gov (NCT03662724).

The data cut-off for this analysis was May 21, 2020. Forty-two patients with AML as defined by the 2016 World Health Organization criteria [7] and a median age of 65 years (range, 24–80 years) had received venetoclax (VEN) with either HMA (*n* = 39) or LDAC (n = 3) and had clinical records about TLS reported. The patient cohort was a high-risk cohort of relapsed (*n* = 18) and refractory (*n* = 22) patients. Two patients received VEN + HMA as frontline therapy. The analysis included 26 patients (62%) with secondary AML and 17 patients (40%) had received prior allogeneic stem cell transplantation. Thirteen patients (31%) had complex cytogenetics, and 37 (88%) had intermediate or poor-risk AML according to ELN 2017 criteria [9]. Overall, 5 patients developed LTLS (12%). Among those, one patient also had elevated creatinine levels and formally met the criteria for clinical tumor lysis. However, this patient already was on dialysis for treatment of chronic end-stage renal disease due to focal segmental glomerulosclerosis before the start of venetoclax. Patients with TLS were younger, more often had de novo AML, less often had been pre-treated with HMAs, more often received voriconazole than posaconazole as antifungal agent, and had higher pre-treatment phosphate and creatinine levels than patients without TLS, while other clinical parameters were similarly distributed (Table 1).

**Table 1:** Patient demographics and baseline characteristics of 42 AML patients with and without TLS

**Baseline characteristics**	**All** **(*n*** **= 42)**	**Without TLS** **(*n =*** **37)**	**With TLS** **(*n =*** **5)**	***P***
Age (years)				0.06
Median (range)	65 (24–80)	68 (24–80)	46 (26–73)	
Sex *n (%)*				0.36
Female	19 (45)	18 (49)	1 (20)	
Male	23 (55)	19 (51)	4 (80)	
Type of AML *n (%)*				< 0.01
De novo	15 (36)	10 (27)	5 (100)	
Secondary	26 (62)	26 (70)	–	
Treatment-related		21	–	
History of MDS		5	–	
Biphenotypic leukemia	1 (2)	1 (3)	–	
ELN classification 2017 *n (%)*				0.67
Favorable	4 (10)	3 (8)	1 (20)	
Intermediate	16 (38)	15 (40)	1 (20)	
Adverse	21 (50)	18 (49)	3 (60)	
Missing	1 (2)	1 (3)	–	
Complex karyotype *n (%)*				0.2
Yes	13 (31)	13 (35)	0	
No	27 (64)	22 (60)	5	
Missing	2 (5)	2 (5)	–	
Treatment lines before VEN				0.84
Median (range)	2 (0–5)	2 (0–5)	2 (1–4)	
WBC at start of VEN (×10^9^/L)				0.47
Median (range)	2.5 (0.1–40.1)	2 (0.1–40.1)	4.8 (0.7–26.3)	
Hemoglobin at start of VEN (g/dL)				0.98
Median (range)	8.8 (6.9–12.3)	8.8 (6.9–12.3)	8.9 (7.2–9.7)	
Platelet count at start of VEN (×10^9^/L)				0.82
Median (range)	33.5 (3–244)	33 (3–244)	65 (6–141)	
Blasts in BM at start of VEN *(%)*				0.18
Median (range)	30 (5–90)	36 (5–90)	25.5 (25–26)	
Blasts in PB at start of VEN *(%)*				0.57
Median (range)	12.4 (0–85)	16 (0–85)	6 (4.6–57)	
AlloHCT before VEN *n (%)*				1.0
Yes	17 (40)	15 (40)	2 (40)	
No	25 (60)	22 (60)	3 (60)	
Previous HMA treatment *n (%)*				0.04
Yes	28 (67)	27 (73)	1 (20)	
No	14 (33)	10 (27)	4 (80)	
Combination partner for VEN *n (%)*				0.51
Azacitidine	27 (64)	25 (68)	2 (40)	
Decitabine	12 (29)	10 (27)	2 (40)	
LDAC	3 (7)	2 (5)	1 (20)	
Azole treatment *n (%)*				0.07
Posaconazole	31 (74)	29 (78)	2 (40)	
Voriconazole	11 (26)	8 (22)	3 (60)	
Pre-treatment calcium mmol/L				0.88
Median (range)	2.15 (1.73–3.28)	2.14 (1.73–3.28)	2.16 (1.93–2.39)	
Pre-treatment potassium mmol/L				0.2
Median (range)	4.1 (3.0–6.1)	4.1 (3.0–5.9)	4.5 (3.2–6.1)	
Pre-treatment phosphate mmol/L				0.03
Median (range)	1.1 (0.54–2.11)	1.08 (0.54–2.11)	1.4 (1.01–1.85)	
Pre-treatment uric acid				0.38
Median (range)	264 (103–444)	285 (103–444)	203 (157–289)	
Pre-treatment creatinine μmol/L				0.01
Median (range)	84.5 (42–843)	81 (42–203)	144 (84–843)	

Dates are n (%) or median (range) unless otherwise noted. *ECOG* Eastern Cooperative Oncology Group, *WBC* white cell count, *BM* bone marrow, *PB* peripheral blood, *AlloHCT* allogeneic hematopoietic cell transplantation

The median day of onset of LTLS was day +2 after the start of chemotherapy (range, 0–7 days). The most frequent laboratory abnormalities were hyperkalemia (4/5, range 6.0–6.3 mmol/L), hyperphosphatemia (3/5, range 1.62–2.06 mmol/L), and hypocalcemia (3/5, range 1.03–1.59 mmol/L). Neither hyperuricemia nor sudden death, arrhythmia, or seizures were recorded in our cohort. Among patients developing TLS, recovery of biochemical values was mostly observed within the first 28 days of chemotherapy (Fig. 1).

**Fig. 1:**
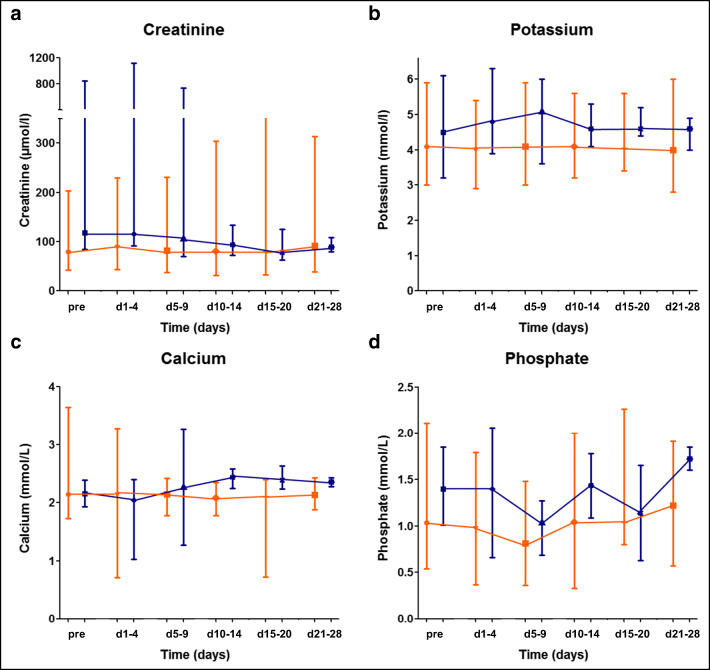
Trends of TLS parameters during the first 28 days of treatment. Graphs showing changes of (**a**) creatinine, (**b**) potassium, (**c**) calcium, and (**d**) phosphate in patients without (*n* = 37, orange) and with (*n* = 5, blue) LTLS (median ± range). Curves for patients with and without TLS are separated for better legibility of the error bars

The median follow-up for patients with TLS was 13.8 months and for patients without TLS 13.5 months. For patients with TLS, the overall response rate (ORR = complete remission (CR) + complete remission with incomplete haematologic recovery (CRi)) was 60% (*n* = 3/5) compared with an ORR of 44% (*n* = 16/37) in patients without TLS (CR/CRi 60% vs. 44%; *p* = 0.48) (Supplementary Table S2). No differences in cumulative incidence of relapse, event-free, and overall survival were observed between patients with and without TLS (Supplementary Table S2 and Supplementary Figs. S1A-C). However, these outcome data are limited by the very small cohort of patients with TLS.

Knowledge about venetoclax toxicity in AML patients has almost entirely been collected from experiences in clinical trials. In clinical trials of treatment naïve AML patients, no events of laboratory or clinical tumor lysis syndrome were observed in the combination of venetoclax plus HMA, while two cases of laboratory (not clinical) TLS were reported with the combination of LDAC plus venetoclax. However, in these trials, patients were required to have WBC count of 25 × 10^9^/L or less at study initiation to mitigate any potential tumor lysis syndrome, with the use of hydroxyurea allowed in order to meet this criterion [4, 10].

We aimed to better understand the risk of TLS in AML patients treated with venetoclax in clinical practice and compare their outcomes with those reported in key clinical trials. In our study, venetoclax treatment in combination with LDAC or HMA without dose ramp-up induced LTLS in 12% of patients, while clinical TLS was formally observed in one dialysis patient only (2%).

LTLS was not associated with clinical characteristics before the start of treatment. Signs of LTLS did not correlate with improved response.

Based on this retrospective analysis, a dose ramp-up for venetoclax does not appear mandatory in AML patients, even when venetoclax is combined with azole antifungals and appropriate reduction of the venetoclax dose. However, the WBC count should be lowered below 25 × 10^9^/L before initiation of venetoclax. Laboratory monitoring on an outpatient basis on day 3 and day 7 after treatment starts appears sufficient in patients without pre-existing signs of TLS or renal failure and WBC below 25 × 10^9^/L. In conclusion, venetoclax dosing without ramp-up is clinically feasible and with consistent efficacy in AML patients even with azole co-medication.

## Electronic supplementary material

ESM 1 (PDF 239 kb)

## References

[1] Bhola PD, Letai A (2016). Mitochondria-judges and executioners of cell death sentences. Mol Cell.

[2] Konopleva M, Pollyea DA, Potluri J, Chyla B, Hogdal L, Busman T, McKeegan E, Salem AH, Zhu M, Ricker JL, Blum W, DiNardo CD, Kadia T, Dunbar M, Kirby R, Falotico N, Leverson J, Humerickhouse R, Mabry M, Stone R, Kantarjian H, Letai A (2016). Efficacy and biological correlates of response in a phase II study of venetoclax monotherapy in patients with acute myelogenous leukaemia. Cancer Discov.

[3] Wei AH et al (2020) Venetoclax plus LDAC for patients with untreated AML ineligible for intensive chemotherapy: phase 3 randomized placebo-controlled trial. Blood. 10.1182/blood.2020004856 10.1182/blood.2020004856 PMC7290090 32219442

[4] DiNardo CD (2019). Venetoclax combined with Decitabine or Azacitidine in treatment-naive, elderly patients with acute myeloid leukemia. Blood.

[5] Heuser M, Ofran Y, Boissel N, Brunet Mauri S, Craddock C, Janssen J, Wierzbowska A, Buske C (2020). Acute myeloid leukaemia in adult patients: ESMO clinical practice guidelines for diagnosis, treatment and follow-up. Ann Oncol.

[6] Roberts AW, Davids MS, Pagel JM, Kahl BS, Puvvada SD, Gerecitano JF, Kipps TJ, Anderson MA, Brown JR, Gressick L, Wong S, Dunbar M, Zhu M, Desai MB, Cerri E, Heitner Enschede S, Humerickhouse RA, Wierda WG, Seymour JF (2016). Targeting BCL2 with Venetoclax in relapsed chronic lymphocytic leukemia. N Engl J Med.

[7] Arber DA, Orazi A, Hasserjian R, Thiele J, Borowitz MJ, le Beau MM, Bloomfield CD, Cazzola M, Vardiman JW (2016). The 2016 revision to the World Health Organization classification of myeloid neoplasms and acute leukaemia. Blood.

[8] Cairo M, Bishop M (2004). Tumour lysis syndrome: new therapeutic strategies and classification. Br J Haematol.

[9] Döhner H, Estey E, Grimwade D, Amadori S, Appelbaum FR, Büchner T, Dombret H, Ebert BL, Fenaux P, Larson RA, Levine RL, Lo-Coco F, Naoe T, Niederwieser D, Ossenkoppele GJ, Sanz M, Sierra J, Tallman MS, Tien HF, Wei AH, Löwenberg B, Bloomfield CD (2017). Diagnosis and management of AML in adults: 2017 ELN recommendations from an international expert panel. Blood.

[10] Wei AH, Strickland SA, Hou JZ, Fiedler W, Lin TL, Walter RB, Enjeti A, Tiong IS, Savona M, Lee S, Chyla B, Popovic R, Salem AH, Agarwal S, Xu T, Fakouhi KM, Humerickhouse R, Hong WJ, Hayslip J, Roboz GJ (2019). Venetoclax combined with low-dose Cytarabine for previously untreated patients with acute myeloid leukemia: results from a phase Ib/II study. J Clin Oncol.

