# Supplementary material for: Risk of tumor lysis syndrome in patients with acute myeloid leukemia treated with venetoclax-containing regimens without dose ramp-up
Source: Ann Hematol. 2020 Jul 23;100(2):595–9. doi: 10.1007/s00277-020-04181-5 (PMC7817590; doi:10.1007/s00277-020-04181-5)
Supplement: Supplementary file 1 — (PDF 239 kb) [file 277_2020_4181_MOESM1_ESM.pdf]

Risk of Tumor Lysis Syndrome in Patients with Acute Myeloid Leukemia Treated with Venetoclax-containing Regimens without Dose Ramp-up

Supporting information

Supplementary methods

Patients and treatment

All patients had received venetoclax in combination with either hypomethylating agents (HMA), namely azacytidine or decitabine or low dose cytarabine (LDAC). Venetoclax was administered at a dose of 100 mg once daily perorally days 1-14 due to mandatory co-medication with a CYP3A4 inhibitor for fungal prophylaxis or treatment, primarily posaconazole or voriconazole. Four patients received venetoclax 100mg days 1-28. Additionally, patients either received azacytidine (n=27, 75 mg/m_2/day subcutaneously days 1-7), decitabine (n=12, 20 mg/m_2/day intravenously days 1-5, two patients received decitabine days 1-10) or low dose cytarabine (n=3, 20 mg/m_2/day twice daily subcutaneously days 1-10, one patient received cytarabine days 1-7). All patients received supportive care measures including uric acid-reducing agents for tumor lysis syndrome, transfusions, hydration and antiemetic agents.

Efficacy and safety assessment

The overall response rate was defined as a combination of complete remission (CR), complete remission with incomplete blood count recovery (CRi) and partial remission (PR) according to ELN 2017 criteria [1]. Morphologic leukemia-free state (MLFS) was defined as less than 5% blasts in an aspirate with a count of at least 200 nucleated cells. Patients were considered as refractory if they did not achieve CR, CRi or PR after four cycles of venetoclax treatment. Overall survival (OS) endpoints, measured from the first day of chemotherapy + venetoclax administration, were death (failure)

and alive at last follow-up (censored). Event-free survival (EFS) endpoints, measured from the first day of chemotherapy + venetoclax administration, were refractory disease (failure), relapse (failure), death from any cause (failure) and alive in CR/CRi at last follow-up (censored). Cumulative incidence of relapse (CIR) endpoints, measured from the date of CR/CRi, were relapse (failure), death from any cause (failure) and alive in CR/CRi at last follow-up (censored) [1].

Patients' charts were searched for laboratory data including levels of creatinine, potassium, calcium, phosphate and uric acid as well as physician-assessed adverse events that emerged during treatment beginning from the first day of venetoclax + chemotherapy administration to day 28 after the start of therapy.

Statistical analysis

Safety and efficacy analyses were performed for all patients who received at least one cycle of venetoclax combination treatment. Demographics were analyzed by descriptive statistics. Median follow-up time for survival was calculated with the reverse Kaplan-Meier method. The Kaplan-Meier method and log-rank test were used to estimate the distribution of OS, CIR and EFS. Comparisons between patients with and without tumor lysis syndrome were conducted using the Wilcoxon-Mann-Whitney-Test test for continuous variables and $\chi 2$ test for categorical variables.

The statistical analyses were performed using Prism software, version 5 (GraphPad Software, Inc., La Jolla, CA, USA), statistical software package SPSS 26.0 (IBM Corporation, Armonk, NY, USA) and Microsoft Excel 2013 (Microsoft Corporation, Redmond, WA, USA).

Supplementary Table S1A. Definition of laboratory tumor lysis syndrome according to Cairo and Bishop [2].

Potassium	≥ 6.0 mmol/L or ≥ 25% increase from baseline
Calcium	≤ 1.75 mmol/L or ≥ 25% decrease from baseline
Phosphate	≥ 1.45 mmol/L or ≥ 25% increase from baseline
Uric acid	≥ 476 µmol/L or ≥ 25% increase from baseline

Laboratory tumor lysis syndrome (LTLS) is defined as either a ≥ 25% change from baseline or level above or below normal, as defined above, for any two or more serum values of potassium, calcium, phosphate, and uric acid within 3 days before or 7 days after the initiation of chemotherapy.

Supplementary Table S1B. Definition of clinical tumor lysis syndrome according to Cairo and Bishop [2].

Creatinine	≥ 1.5 fold ULN*
Cardiac arrhythmia/sudden death	Not directly or probably attributable to a therapeutic agent
Seizure	Not directly or probably attributable to a therapeutic agent

* Creatinine levels: patients will be considered to have elevated creatinine if their serum creatinine is 1.5 fold greater than the institutional upper limit of normal (ULN). Clinical tumor lysis syndrome (CTLS) is defined as the presence of LTLS and any one or more of the above-mentioned criteria. ULN, upper limit of normal.

Supplementary Table S2. Response and outcome analysis in AML patients with and without TLS.

	**Without TLS (n=37)**	**With TLS (n=5)**	***P***
Best response *n (%)*			0.77
ORR (CR, CRi)	16 (44)	3 (60)	
CR/CRi	16 (44)	3 (60)	
MLFS	3	-	
SD/RD/PD	11 (30)	2 (40)	
Death before assessment	6 (16)	-	
Pending	1 (3)	-	
Follow-up (months)			
Median	13.5	13.8	
Event-free survival (months)			0.63
Median (95% CI)	4.3 (1.1-7.3)	6.7 (1.9-11.6)	
Cumulative incidence of relapse (months)			0.41
Median (95% CI)	8.0 (5.4-10.6)	4.5 (1.3-7.7)	
Overall survival (months)			0.058
Median (95% CI)	5.3 (2.3-8.4)	NR	

Data are n (%) or median (95% CI) unless otherwise indicated. Patients with stable disease or progressive disease were considered non-responders. ORR, overall response rate; CR, complete remission; CRi, complete remission with incomplete hematologic recovery; MLFS, morphologically leukemia free state; SD, stable disease; RD, refractory disease; PD, progressive disease; CI, confidence interval; NR, not reached.

Supplementary Figures

Figure S1. Survival analysis of AML patients with and without TLS.

(A) Event-free survival of patients with TLS (n=5, blue line) and without TLS (n=37, orange line).

(B) Cumulative incidence of relapse of CR/CRi patients with TLS (n=3, blue line) and without TLS (n=16, orange line).

(C) Overall survival of patients with TLS (n=5, blue line) and without TLS (n=37, orange line).

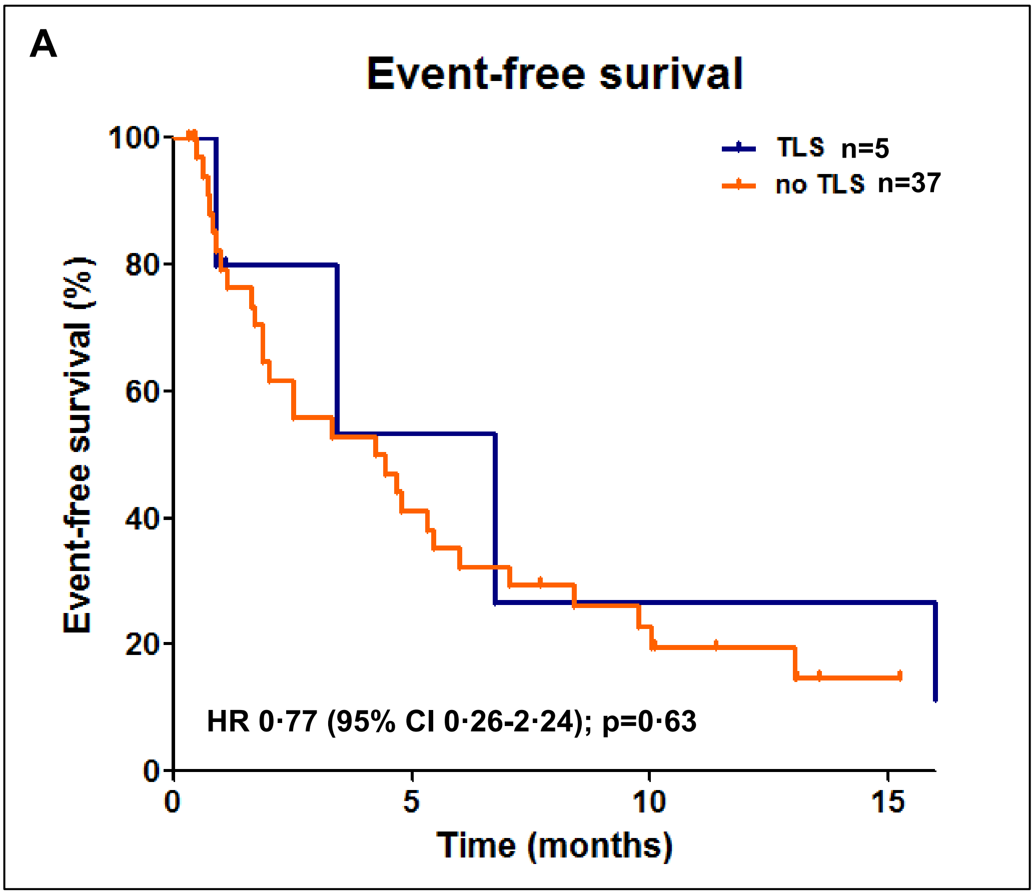

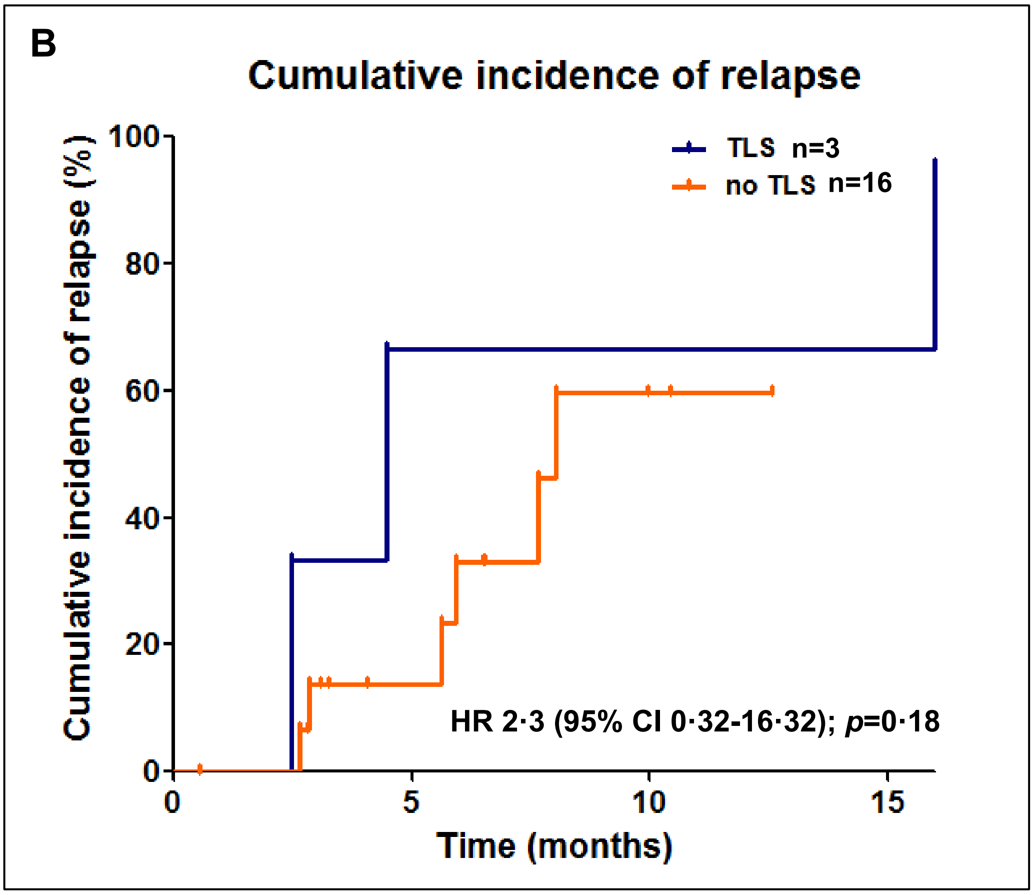
B
Cumulative incidence of relapse
Cumulative incidence of relapse (%)
100
80
60
40
20
0
TLS n=3
no TLS n=16
HR 2·3 (95% CI 0·32-16·32); p=0·18
0
5
10
15
Time (months)

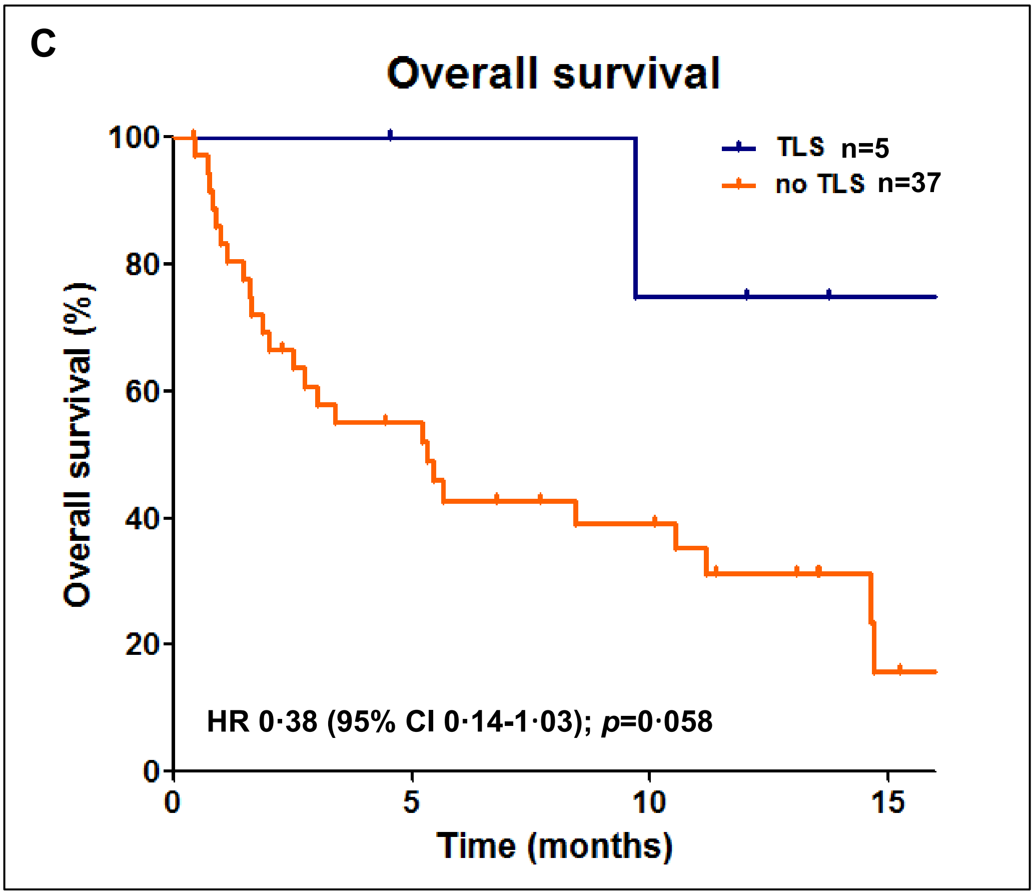
C
Overall survival
Overall survival (%)
100
80
60
40
20
0
TLS n=5
no TLS n=37
HR 0·38 (95% CI 0·14-1·03); p=0·058
0
5
10
15
Time (months)

References

1. Döhner H et al (2017) Diagnosis and management of AML in adults: 2017 ELN recommendations from an international expert panel. Blood 129(4):424–447. https:// doi:10.1182/blood-2016-08-733196

2. Cairo MS, Bishop M (2004) Tumour lysis syndrome: new therapeutic strategies and classification. Br J Haematol 127:3-11. https://doi:10.1111/j.1365-2141.2004.05094.x
